# Supplementary material for: Chromothripsis during telomere crisis is independent of NHEJ, and consistent with a replicative origin
Source: Genome Res. 2019 May;29(5):737–49. doi: 10.1101/gr.240705.118 (PMC6499312; doi:10.1101/gr.240705.118)
Supplement: Supplemental Material [file supp_gr.240705.118_Supplemental_file_1.zip › contigs/annotated_contigs/DB75/contig.2.DB75_length_489_mean_cov_6.30470347648.docx]

**DB75_length_489_mean_cov_6.30470347648**

```
TTTTGATTCATAAAAGAGTTTCATTAATACTAGATATTTCAGGAAATTATCCTTGACTAATACGAGGGATGGAAGGAATGGGGGAAAAC
 >chr7:126708015-126708230 - E=8e-118 p=1e-02
TCAAGGCTCACCATATCATGGTAAATTATTTATTTATTGTTTATTTAGTGGGAAAGGATCTGCTCTGTTGTGTGCTTTCACTGGTCTAA

AGGGATTTGGCACAGTTACCTAAATAGGTGTAATTAT|TGTATTACCTGACATAGTGCTGGCCCATAGTTAAATACATGAATTCATGAA
                                       >chr7:126735851-126736125 - E=2e-153
ATACCAGTCAGAAATGGCTCACCAAATCTGCTTGCTTCCTTTTTCATTTTGAATCTTCTTAAGAATGAATCAATTCAGTGAGCATTTAT

AGTGCATGTGTCAGGTGATACTTGTGCTGGGCTTTGAGGATACAGAGACAAATCATGGTCTTTGCTTTCCAGCAGGTCACTGTGTTTCA

GAGGAGGTGGAGATGCAAACAATTACAAATTCATGTGATCTTTGC
```
